# Development of a primary cell model derived from porcine dorsal soft palate for foot-and-mouth disease virus research and diagnosis

**DOI:** 10.3389/fmicb.2023.1215347

**Published:** 2023-09-29

**Authors:** Morgan Sarry, Cindy Bernelin-Cottet, Caroline Michaud, Anthony Relmy, Aurore Romey, Anne-Laure Salomez, Patricia Renson, Maud Contrant, Maxime Berthaud, Hélène Huet, Grégory Jouvion, Sara Hägglund, Jean-François Valarcher, Labib Bakkali Kassimi, Sandra Blaise-Boisseau

**Affiliations:** ^1^UMR VIROLOGIE, INRAe, EnvA, ANSES Laboratoire de Santé Animale, Université Paris-Est, Maisons-Alfort, France; ^2^AgroParistech, Paris, France; ^3^ANSES Laboratoire de Ploufragan-Plouzané-Niort, Ploufragan, France; ^4^Dynamyc Research Team, Université Paris-Est Créteil, Ecole Nationale Vétérinaire d’Alfort, ANSES, Créteil, France; ^5^Unité d’Histologie et d’Anatomie Pathologique, Ecole Nationale Vétérinaire d’Alfort, Maisons-Alfort, France; ^6^Host Pathogen Interaction Group, Section of Ruminant Medicine, Department of Clinical Science, Swedish University of Agricultural Sciences (SLU), Uppsala, Sweden

**Keywords:** foot-and-mouth disease virus (FMDV), FMDV persistence, cellular model, primary cells, swine

## Abstract

Foot-and-mouth disease (FMD) is a highly contagious viral disease of cloven-hoofed animals that has a significant socio-economic impact. One concern associated with this disease is the ability of its etiological agent, the FMD virus (FMDV), to persist in its hosts through underlying mechanisms that remain to be elucidated. While persistence has been described in cattle and small ruminants, it is unlikely to occur in pigs. One of the factors limiting the progress in understanding FMDV persistence and, in particular, differential persistence is the lack of suitable *in vitro* models. A primary bovine cell model derived from the dorsal soft palate, which is the primary site of replication and persistence of FMDV in cattle, has been developed, and it seemed relevant to develop a similar porcine model. Cells from two sites of FMDV replication in pigs, namely, the dorsal soft palate and the oropharyngeal tonsils, were isolated and cultured. The epithelial character of the cells from the dorsal soft palate was then assessed by immunofluorescence. The FMDV-sensitivity of these cells was assessed after monolayer infection with FMDV O/FRA/1/2001 Clone 2.2. These cells were also grown in multilayers at the air-liquid interface to mimic a stratified epithelium susceptible to FMDV infection. Consistent with what has been shown *in vivo* in pigs, our study showed no evidence of persistence of FMDV in either the monolayer or multilayer model, with no infectious virus detected 28 days after infection. The development of such a model opens up new possibilities for the study and diagnosis of FMDV in porcine cells.

## 1. Introduction

Foot-and-mouth disease (FMD) is a highly contagious transboundary disease affecting wild and domestic cloven-hoofed animals. It is one of the most important animal diseases because of its socio-economic impact in case of an outbreak (Knight-Jones and Rushton, 2013). The etiological agent of FMD, known as foot-and-mouth disease virus (FMDV), is a single-stranded positive RNA virus that belongs to the genus *Aphtovirus* within the *Picornaviridae* family (Jamal and Belsham, 2013). FMDV has a high mutation rate, resulting in the existence of seven different serotypes, namely, A, O, C, Asia-1, Southern African Territories (SAT) 1, SAT2, and SAT3, and many lineages and sublineages (Knowles and Samuel, 2003). The FMDV genome contains an 8.5 kb open reading frame (ORF), flanked by untranscribed regions (5′ UTR and 3′ UTR). It encodes a single polyprotein precursor composed of four structural proteins (VP1, VP2, VP3, and VP4) and 11 non-structural proteins (Lab_pro_, Lb_pro_, 2A, 2B, 2C, 3A, 3B1, 3B2, 3B3, 3C, and 3D^pol^; Belsham et al., 2020).

The clinical signs induced by FMDV are characterized by fever and the development of vesicles or aphthae on multiple mucosa and epithelia (tongue, legs, muzzle, teats, mammary gland, prepuce, vulva, and other parts of the skin), a state of lethargy, and loss of appetite (Kitching, 2002). The clinical signs are quite easily noted in domestic cattle, which are considered to be revelatory, but less marked in small ruminants, which act as disseminators, and in African buffalo, in which the infection is mild or subclinical. Swine, considered amplifiers, show severe clinical signs often characterized by lameness, a withdrawn posture, and reluctance to stand or walk as a direct consequence of podal lesions. While mortality in adult animals is generally low, it can be high in young animals due to acute myocarditis, particularly in piglets, lamb, and calves (Stenfeldt and Arzt, 2020). The severity of clinical signs in susceptible species may vary according to the specific virulence of FMDV strains. Indeed, some mutations affecting the FMDV 3A protein, in particular, can affect the host range. For instance, the O/TAW/97 strain caused severe lesions in swine, while no cases were observed in ruminants (Beard and Mason, 2000; Knowles et al., 2001).

After clinical recovery, FMDV persists in up to 50% of ruminants. These become carriers without clinical signs, regardless of their specific FMD immune status [Arzt et al., 2011a; World Organization for Animal Health (OIE), 2021]. Such healthy carriers represent a potential threat of transmission of FMDV to susceptible animals, a source of new recombinants, and remain thus an obstacle to FMDV control (Arzt et al., 2018; Stenfeldt and Arzt, 2020; Childs et al., 2022; Fish et al., 2022). FMDV persistence was originally defined as an infectious virus detected beyond 28 days postinfection. This arbitrary threshold is challenged by the fact that viral clearance has been shown to occur earlier than previously assumed, between 10 and 21 days beyond infection depending on the animal vaccination status (Stenfeldt and Belsham, 2012; Stenfeldt et al., 2016a; Stenfeldt and Arzt, 2020). More than 50 years after it was first described, the mechanisms of establishment, maintenance, and resolution of FMDV persistence are still not fully understood (Van Bekkum et al., 1959). Among the existing gaps of knowledge, the differential persistence of FMDV is also not explained. For example, there are currently no data to understand why FMDV persistence has been reported in cattle and small ruminants, but not in pigs (Burrows, 1968; Stenfeldt et al., 2016a). Over the last decades, the primary sites of infection as well as the sites of FMDV persistence in different susceptible species have been located. Domestic cattle and buffalo persistent sites mainly consist in their dorsal soft palate and dorsal nasopharynx (Arzt et al., 2011b). The dorsal soft palate, as well as palatine tonsils, are the persistent sites in sheep and goats (Arzt et al., 2011a). In swine, which are more susceptible to infection via exposure of the upper gastrointestinal tract than to infection through inhalation of the virus, specialized epithelium within porcine oropharyngeal tonsils has been described as the primary site of FMDV replication, while epithelium of the soft palate tonsils hosts significant replication of the virus during the clinical infection phase (Sellers and Gloster, 2008; Stenfeldt et al., 2014, 2016c). While persistent infectious viruses, as well as FMDV proteins and RNA, are detected in the persistence sites of carrier ruminants, only long-lasting FMDV proteins and RNA are frequently detected after infection in the swine FMDV replication sites (Arzt et al., 2011a,b; Stenfeldt et al., 2016b).

One limiting factor in the progress of knowledge about FMDV persistence is the lack of suitable models. Indeed, studies performed on animals require infrastructures that only a few laboratories worldwide have, as well as being costly and raising ethical issues. Since the development of *in vitro* techniques, allowing the growth of the virus, various epithelial cell lines have served as study models, such as Baby Hamster Kidney 21 (BHK-21), Instituto Biologico-Rim Suino-2 (IBRS-2), Porcine Kidney (PK-15), Swine Kidney (SK-6), Fetal porcine kidney (LFBK-*α_V_β6*), Fetal goat tongue (ZZ-R127), and Madin-Darby Bovine Kidney (MDBK) cells (De Castro, 1970; Kasza et al., 1972, p. 6; De La Torre et al., 1985; Kopliku et al., 2015; Gray et al., 2020). Except for the ZZ-R127 cells, none of these lines were derived from FMDV-sensitive tissue, which raises the question of a possible bias in the results obtained. The use of relevant primary cells may overcome such bias. Primary bovine thyroid (BTY) cells are, for example, among the reference cells for the study of FMDV. However, primary BTY cells cannot be passaged in a stable manner or frozen with remaining sensitivity. This problem was overcome with the development of a BTY-derived cell line (Mao et al., 2018). Primary cells, like cell lines, share a drawback, which is the need to be regularly passed to survive. To avoid such cell passages, a multilayer cellular model derived from primary bovine dorsal soft palate (DSP), cultured at the air-liquid interface (ALI), has been developed (Hägglund et al., 2020). We have shown that this *in vitro* DSP-ALI model enabled the establishment of a persistent FMDV infection and has led to improved knowledge concerning transcriptional responses following FMDV infection (Pfaff et al., 2019).

Given the opportunities offered by such a model and the absence of a suitable porcine cell model, we isolated primary cells from porcine oropharyngeal tonsils and dorsal soft palate in order to develop a multilayer culture model at the air-liquid interface. We tested if these cells were expressing epithelial markers, checked their ability to produce type I interferons and activate interferon-stimulating genes, and assessed their sensitivity to a panel of FMDV strains of different serotypes and to other viruses that induce similar lesions as FMDV. We then studied the infection by FMDV of these cells as monolayers and multilayers.

## 2. Materials and methods

### 2.1. Virus

The FMDV O/FRA/1/2001 Clone 2.2 (GenBank: OV121130.1) used in this study is a twice-plaque-purified viral clone derived from the O/FRA/1/2001 strain that was further propagated on BHK-21 cells (Kopliku et al., 2015). Viral batches were titrated on BHK-21 monolayers by plaque assay as described in a previous study (Kopliku et al., 2015). Viral titer is equal to 1.4 × 10^6^ Plaque Forming Unit (PFU) per mL.

Viruses used for assessment of porcine DSP susceptibility to FMDV and look-alike disease viruses were previously grown in IBRS-2 cell monolayers, and lysates were clarified by centrifugation and stored at −80°C. A panel of eight FMDV strains representative of field viruses recently detected, namely, Asia1/PAK/2011, SAT1/NIG/2015, SAT3/ZIM/1981, O/OMN/2020, O/FRA/2001, O/MUR/2016, O/ALG/2018, and A/TUN/2017, and a panel of two strains of Vesicular Stomatitis Virus (VSV) IND/1942 and NJ/1964, Seneca Valley Virus (SVV) CA/2001 and MN/1988, as well as Swine Vesicular Disease Virus (SVDV) FRA/1973 and ITL/2008, were tested.

### 2.2. Viral titration

Foot-and-mouth disease virus titration by TCID50 assays was performed as described by Hägglund et al. (2020). VSV, SVV, and SVDV titration by TCID50 assays were performed similarly to FMDV titrations but using porcine DSP cells.

### 2.3. Isolation of cells from the swine dorsal soft palate

Epithelial tissue from the DSP was collected immediately after the slaughter of three American Yorkshire swine (id: 100–309, 100–310, and 100–313) that were aged 4–6 months. They had undergone anti-basal glomerulus lamp treatment, which was considered to have no impact on the oropharyngeal structures of interest. During transport to the FMDV laboratory, located on the same site, tissues were stored in sterile containers with transport medium consisting of Dulbecco’s Modified Eagle’s Medium (DMEM, Lonza, Belgium), supplemented per liter with 2.5 mg amphotericin B (Sigma-Aldrich, A-9528) and 20 mg gentamicin (Sigma-Aldrich, G-1397). Dissociation of the surface epithelium was performed to remove as much connective and muscle tissue as possible. Epithelial tissue was dissected and digested at 4°C overnight in an incubation medium composed of DMEM supplemented per liter with 2.5 mg amphotericin B, 1 mg deoxyribonuclease (Sigma-Aldrich, DN-25), 1 g dithiothreitol (Sigma-Aldrich, D-0632), 20 mg gentamicin, 20 mL 1 M HEPES (VWR, BioWhittaker BE17-737F), 10 mL 200 mM L-glutamine (VWR, BioWhittaker BE17-605F), and 100 mg penicillin–streptomycin (PS, Invitrogen), and additionally supplemented per liter with 1 g protease XIV (Sigma-Aldrich, P5147).

Epithelial cells were thereafter manually scraped off the underlying tissue, filtered through a 40 μm cell strainer (Corning), and incubated in cell culture flasks for 4 h at 37°C in a 5% CO_2_ atmosphere with culture medium to deplete the fibroblasts. This medium consisted of DMEM containing 10% gamma-irradiated, heat-inactivated, fetal calf sera (FCS, Hyclone™, GE Healthcare) and supplemented per liter with 31.25 KU Nystatin (Sigma-Aldrich, N6261), in addition to HEPES, L-glutamine, and PS as above. The epithelial cells were centrifuged at 200 × *g* for 10 min at room temperature and cultured at 37°C in a 5% CO_2_ atmosphere in flasks containing culture medium.

### 2.4. Tissue histological staining

After necropsy, tissue samples from the oropharyngeal tonsils and dorsal soft palate were removed and immediately fixed in neutral-buffered formalin for 48 h. Samples were then embedded in paraffin; 4 μm-thick sections were cut and stained in hematoxylin–eosin-saffron (HES).

### 2.5. Air-liquid interface multilayers

Coated inserts with a 12 mm diameter and 1.0 μm pores (cellQART® 12-Well Insert 1.0 μm PET clear, SABEU) were rehydrated by soaking in a 12-well plate containing 1 mL of DMEM high glucose W/L-GLU W/O PY (Eurobio, L0102)-based complete culture medium (10% FCS, 2% HEPES, 1% L-glutamine, 1% PS, and 0.2% gentamicin) and by adding 1 mL of this medium directly on the inserts. The inserts were left in contact with the medium for 2 h at 37°C in a 5% CO_2_ atmosphere. Dorsal soft palate cells previously propagated in cell culture flasks were then seeded on the rehydrated inserts at a density of 7.5 × 10^5^ cells per insert in 1 mL of DMEM high glucose-based complete culture medium supplemented with 0.02 μg/mL recombinant human hepatocyte growth factor (HFG, Sigma-Aldrich, H9661). As the cells formed a complete monolayer, the culture medium was removed from the upper compartment after 4 days of culture. The culture medium contained in the lower compartment was changed every 2 or 3 days for 5 weeks.

### 2.6. Cell characterization

The cellular expression of epithelial markers, such as cytokeratin, vimentin, and occludin, as well as that of integrin α_V_β_6_, the specific receptor for FMDV, was analyzed after cell passages in culture flasks, as well as in the cell multilayers cultured on inserts at ALI. For monolayer characterization, cells were cultured in a 8-well μ-Slide (high ibiTreat 1.5 polymer coverslip, Ibidi). Once the cells had adhered to the support, they were fixed in 4% paraformaldehyde (PFA, Electron Microscopy Sciences) for 30 min at room temperature and permeabilized in 0.1% Triton X-100. Cell nuclei were stained using DAPI (Life Technologies) according to the manufacturer’s instructions. Proteins of interest were detected by immunofluorescence microscopy using a DMi8 microscope (Leica Microsystems). A cocktail composed of two mouse monoclonal antibodies (clones A1/A3, DAKO, M3515), which recognize cytokeratins 1, 2, 3, 4, 5, 6, 7, 8, 10, 13, 14, 15, 16, and 19, was used to stain cytokeratin. Vimentin staining was performed using a mouse monoclonal antibody (Clone LN-6, Sigma-Aldrich, V2258). Occludin staining was performed using a rabbit polyclonal antibody (Affinity, DF7504), and a mouse monoclonal antibody directed against integrin α_V_β_6_ (clone 10D5, Abcam, ab77906; Burman et al., 2006) was used to stain integrin α_V_β_6_. Immunofluorescence (IF) was performed using two secondary antibodies: anti-mouse Alexa 488 (Invitrogen) for integrin, vimentin, and cytokeratin revelation, and anti-rabbit Alexa 488 (Invitrogen) for occludin revelation.

### 2.7. Multilayers characterization

The cell morphology was studied by light after fixation in 10% buffered formalin. Selected multilayers and the underlying PTFE membranes were embedded in 1.3% agarose and then left in 70% ethanol overnight. They were then embedded in paraffin, routinely processed, sliced at 4 μm, stained with hematoxylin–eosin-saffron (HES), and examined by light microscopy.

### 2.8. Detection of common swine viruses

In order to detect DNA and RNA from viruses frequently circulating in French swine populations, such as porcine reproductive and respiratory syndrome (PRRS) and porcine circovirus 2 and 3 (PCV2 and PCV3), molecular detection tests were performed using RNA and DNA from the culture supernatant and from DSP cell pellets. RNA and DNA extraction was performed using the NucleoSpin virus kit (Macherey-Nagel). Pellets were resuspended in 700 μL of RAV1 lysis buffer, then ground with stainless steel beads for 30 s at 25 Hz before centrifugation. A total of 600 μL of shred supernatant was then incubated with proteinase K for 1 min at room temperature, vortexed, and incubated at 70°C for 5 min. A further extraction was carried out according to the supplier’s recommendations. Porcine reproductive and respiratory syndrome virus detection was attempted by using quantitative PCR using RT-PCR VetMAX PRRSV EU&NA 2.0 kit (ThermoFisher) and CFX96 Touch Real-Time PCR Detection System (BioRad), according to the supplier’s recommendations. Porcine circovirus 2 detection was attempted by using quantitative PCR searching for the CAP gene. The PCR reaction was performed in a 25 μL volume, with 1X UMM2X, 100 nM of each primer (forward: 5′-GGCGGTGGACATGATGAG-3′ and reverse: 5′-GGGAGCAGGGCCAGAATT-3′), 200 nM of the TaqMan FAM-MGB/NFQ probe (5′-ACCTTAACCTTTCTTATTCTG-3′), and 5 μL of sample DNA. PCR conditions were as follows: 2 min at 50°C, 10 min at 95°C, followed by 40 cycles of 15 s of denaturation at 95°C and 1 min of hybridization and elongation at 60°C. Porcine circovirus 3 detection was attempted by using quantitative PCR searching for the REP gene (Franzo et al., 2018). The PCR reaction was performed in a 25 μL volume, with 1X UMM2X, 300 nM of each primer (forward: 5′-CGGCGGGAAATCTGACTGAA-3′ and reverse: 5′-TACCCAACCCCATCACCCC-3′), 200 nM of the TaqMan FAM-MGB/NFQ probe, and 5 μL of sample DNA. PCR conditions were the same as above.

### 2.9. FMDV inoculation of cell monolayers

**Figure 1:**
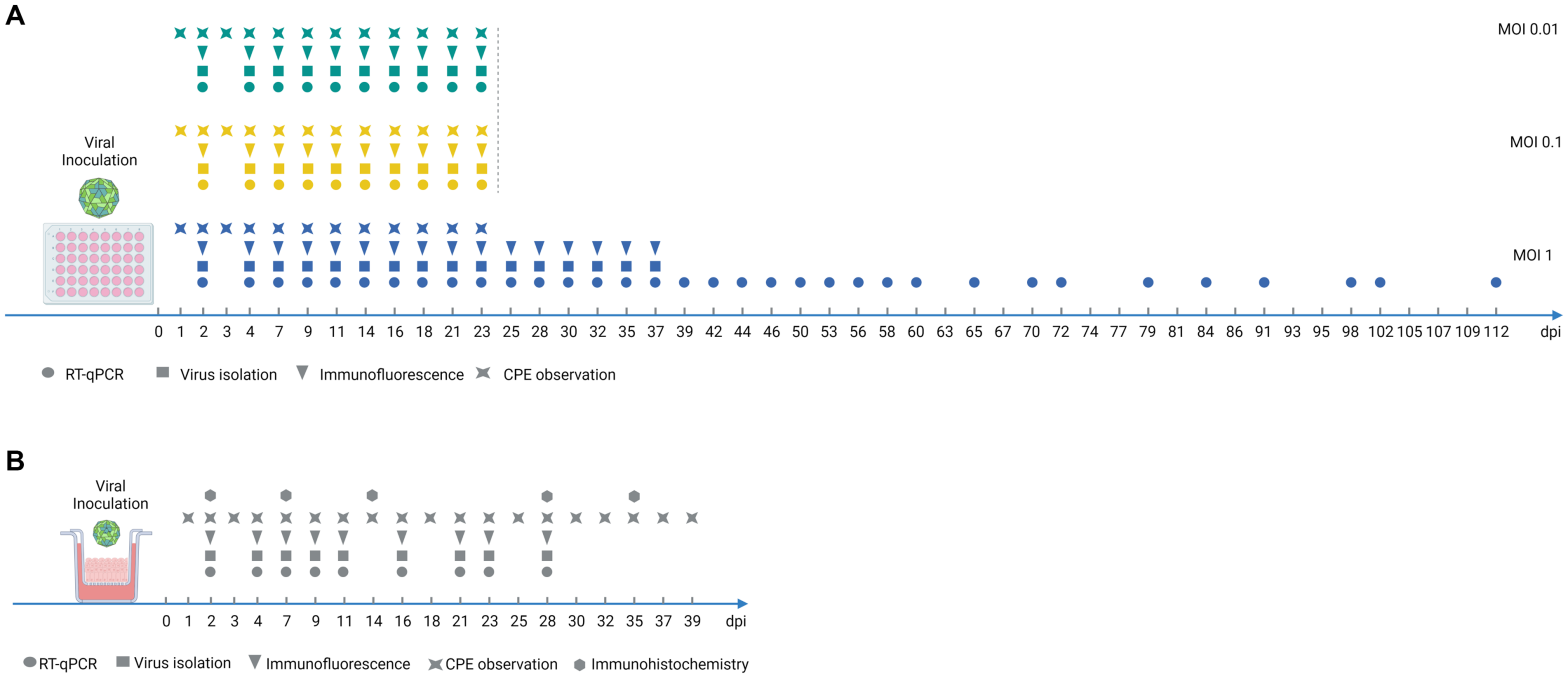
Experimental timeline for monitoring FMDV infection of porcine DSP. **(A)** Experimental timeline for monitoring FMDV infection of porcine DSP cultured in monolayers. Porcine DSP cells were propagated in monolayers and were infected with FMDV O Cl2.2 at MOI 0.01, 0.1, or 1, or a placebo-conditioned medium. Culture supernatants were collected every 2 or 3 days during 112 dpi as illustrated above. **(B)** Experimental timeline for monitoring FMDV infection of porcine DSP cultured in multilayers at the air-liquid interface. Porcine DSP cells were propagated in multilayers on porous membrane inserts at the air-liquid interphase and were infected with FMDV O Cl2.2 at MOI 1, or a placebo-conditioned medium. Insert upper supernatants were collected every 2 or 3 days during 39 dpi as illustrated above. Analyses were performed on collected supernatants: virus isolation (squares) and RT-qPCR (circles) immunofluorescence (triangles). Immunochemistry analyses were performed on fixed inserts (hexagons) and CPE observations were also performed (stars), at indicated days postinfection.

Dorsal soft palate cells were propagated in culture flasks for five passages before being seeded in 48-well plates at a density of 1.0 × 10^5^ cells per well in a DMEM high glucose complete medium. Once the cells formed a complete monolayer, two wells were trypsinized in order to count the cells and adjust the multiplicity of infection (MOI). The viral inoculum of FMDV O/FRA/1/2001 Clone 2.2 was diluted at MOI 1, MOI 0.1, and MOI 0.01 in a serum-free DMEM high glucose complete medium. A total of 100 μL of viral inoculum or 100 μL of conditioned medium (MOCK) was added to the corresponding wells. After 1 h, 250 μL of DMEM high glucose complete medium was added to each well. Visual estimation of the cytopathic effect (CPE) from 0% (no destruction) to 100% (no more adherent cells) was performed daily during the first week postinfection to evaluate the viral impact on the cells. Every 2 or 3 days, the supernatants were collected and stored for further investigations as described in Figure 1A, and a fresh DMEM high glucose complete medium was added. When the cells became confluent, they were trypsinized and seeded into a larger well.

### 2.10. FMDV inoculation of multilayered cells

Dorsal soft palate cells were cultured on inserts for 5 weeks and were thereafter inoculated with FMDV O/FRA/1/2001 Clone 2.2 at MOI 1 or incubated with a conditioned medium. Visual estimation of the cytopathic effect (CPE) was performed daily during the first week postinfection. Two days post-infection (dpi) the viral inoculum or the conditioned medium contained in the upper compartment was harvested and the upper part of the insert was let dry. Every 2 or 3 days, a fresh DMEM high glucose complete medium was added to wash the inserts and immediately collected for further investigations as described in Figure 1B, and the culture medium contained in the lower compartment was renewed. Inserts were regularly harvested and fixed, embedded, and then cross-sectioned for further staining.

### 2.11. Type-I IFN response investigation

Porcine DSP and bovine DSP were cultured in 6-well plates at a density of 1.5 × 10^6^ cells per well in a DMEM high glucose complete medium. After 24 h, half of the wells were infected by FMDV O/FRA/1/2001 Clone 2.2 (MOI 1 for porcine DSP and MOI 0.01 for bovine DSP) and the other half were incubated with conditioned medium as described above. After 6, 12, and 24 h postinfection, cells were collected in order to extract RNA. Extracted RNA was used to perform five simplex rtRT-PCR targeting bovine and porcine IFNα, IFNβ, MX-1, PKR, and β-actin housekeeping gene, using AgPath-ID One-Step RT-PCR kit (Applied Biosystems) and following suppliers recommendations. The primers used were adapted from O’Donnell et al. (2014).

### 2.12. Detection of infectious FMDV by viral isolation

Porcine kidney epithelial cells (IBRS-2, CCLV-RIE 103, and FLI) were cultured in Earle’s essential minimum medium with L-glutamine, supplemented with 7% FCS, 1.5% lactalbumin hydrolysate (SigmaAldrich), 1% PS, and 25 mM HEPES. Then, 96-well plates were seeded with 5 × 10^4^ IBRS-2 cells per well and incubated at 37°C in a 5% CO_2_ atmosphere for 24 h. The monolayers were then washed twice with serum-free culture medium and were inoculated with 50 μL of upper supernatant. One hour later, 250 μL of culture medium was added. The cells were then incubated at 37° C in a 5% CO_2_ atmosphere for 48 h. CPE was visually monitored at 24 and 48 h post-infection (hpi).

### 2.13. Detection of FMDV 3D^pol^ antigen by immunofluorescence

Foot-and-mouth disease 3D^pol^ antigen detection was performed 48 h post virus inoculation on IBRS-2 cells, by IF. The 96-well plates were fixed with 4% paraformaldehyde (Electron Microscopy Sciences) for 25 min, then permeabilized with Triton X-100 for 30 min. 3D^pol^ staining was performed using mouse monoclonal antibody 3F12 anti-FMDV 3D^pol^ at 1:500 (kindly provided by Dr. Emiliana Brocchi and Dr. Santina Grazioli of IZSLER, Brescia, Italy) and goat anti-mouse AlexaFluor 488 IgG H + L (Life Technologies) was used at 1:1,000. Cell nuclei were stained using DAPI (Life Technologies) according to the manufacturer’s instructions. Positivity for FMDV antigen was assessed by IF observation using a DMi8 microscope (Leica Microsystems). The observations were classified according to an arbitrary index ranging from 0 to 3 based on visual estimation of the proportion of cells in which 3D^pol^ antigen is detected: 0 means no detection, 1 indicates the presence of a few 3D^pol^ antigen-positive cells, 2 represents wells in which more than half of the cells are positive, and 3 indicates that almost all cells are 3D^pol^ antigen-positive.

### 2.14. Detection of FMDV RNA by real-time RT-PCR

Viral RNA was extracted from 100 μL of cell culture supernatants using the ID Gene Mag Universal kit (Innovative Diagnostic) and a KingFisher Flex automat, according to the supplier’s recommendations in an elution volume of 60 μL. Molecular FMDV detection was performed by real-time (rt) RT-PCR targeting 3D^pol^ gene, as well as GAPDH housekeeping gene (Bernelin-Cottet et al., in prep.) using AgPath-ID One-Step RT-PCR kit in a final volume of 25 μL. For each PCR, 12.5 μL of buffer 2X, 1 μL of RT-PCR enzyme mix 25X according to suppliers’ recommendations, and each primer and TaqMan probe at 0.2 μM were mixed with 5 μL of sample RNA. PCR amplifications were performed with a QuantStudio™5 Real-Time PCR Instrument (Life Technologies) for 10 min at 45°C, for 10 min at 95°C followed by 45 cycles composed of 15 s at 95°C, and for 1 min at 60°C. Results were analyzed with the *QuantStudio Design & Analysis software* v1.5.1 (Thermo Fisher Scientific).

## 3. Results

### 3.1. Porcine DSP cells are *in vitro* cultivatable epithelial cells

**Figure 2:**
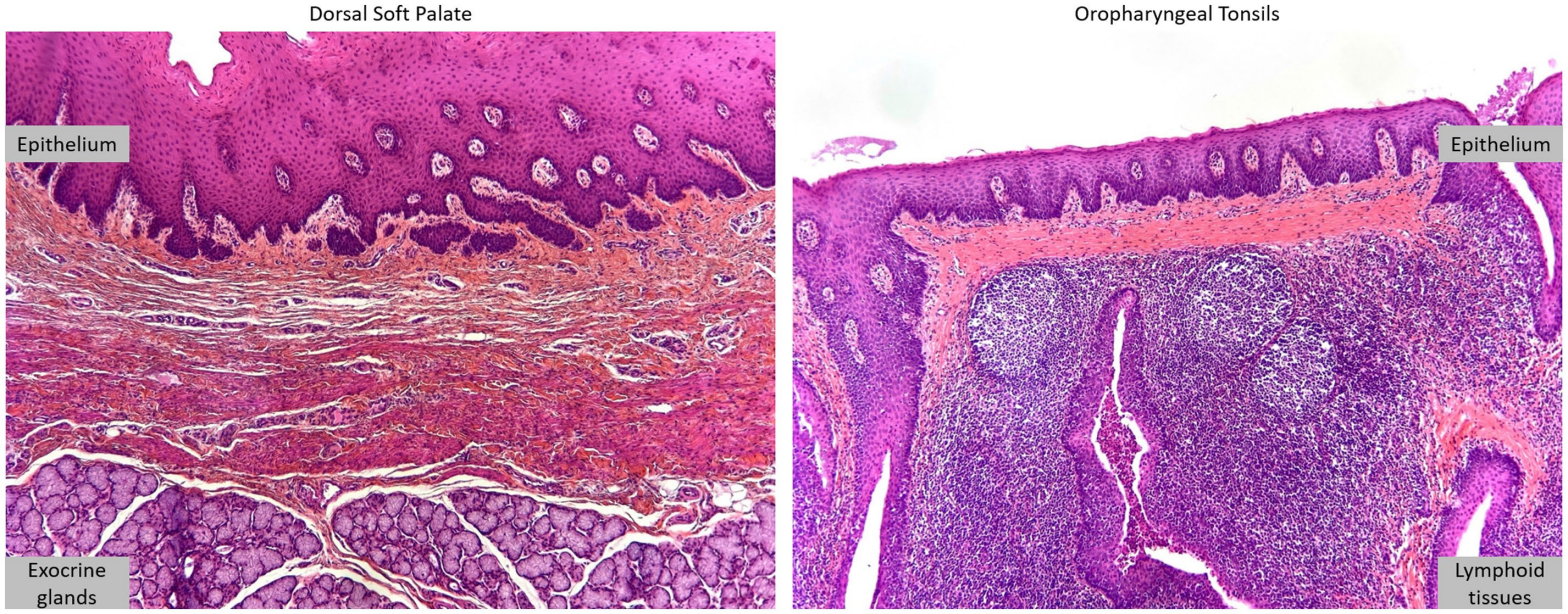
Oropharyngeal tonsils and dorsal soft palate. DSP and oropharyngeal tonsils were collected from 4 to 6-month-old American Yorkshire swine. Histological analysis, HES staining.

Histological analysis of oropharyngeal tonsils and DSP revealed epithelial structures associated with lymphoid tissue and exocrine glands, respectively (Figure 2).

This analysis confirmed that the samples were taken from the correct area with an intact epithelium for cell isolation. Primary cells from the oropharyngeal tonsils of three swine (100–309, 100–310, and 100–313) were isolated but only a few adhered to the culture plates. These cells did not survive the first trypsinization and could not be further propagated. For the primary DSP cells, the dorsal soft palates from animals 100–309 and 100–310 were not treated because the dorsal soft palates were slightly damaged during collection. Thus, only the dorsal soft palate from swine 100–313 was used to isolate primary cells. After initial sub-optimal passages in complete DMEM (slow growth and difficulty in detaching cells), culture in complete DMEM high glucose significantly improved culture conditions and enabled cell characterization, as well as infection assays in monolayers and multilayers. Thus, only the DSP from swine 100–313 was used for further experimentation.

In order to ensure that the primary cultured cells did not carry other viruses, we performed PCR tests, using RNA from the culture supernatant and from DSP cell pellets, for the detection of viruses that are frequently circulating in French swine populations and that may persist in the long term. No viral RNA nor DNA from PRRS, PCV2, or PCV3 was detected.

**Figure 3:**
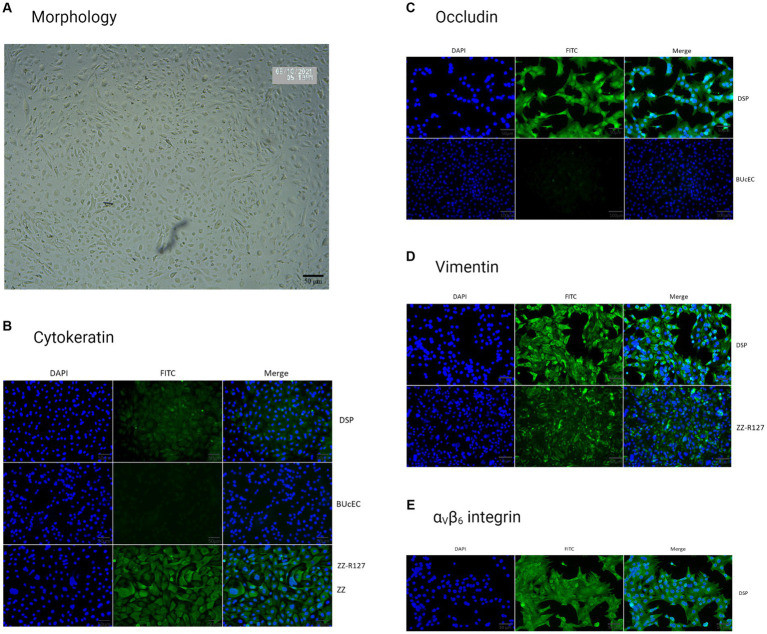
Porcine DSP cells characterization. **(A)** Porcine DSP morphological observation. DSP cells were cultured in monolayers and were observed under brightfield microscopy (x100). **(B)** Cytokeratin staining. DSP was cultured in monolayers on High ibiTreat Ibidi chamber slides. Cell nuclei were stained with Hoechst (blue) and cells were visualized by fluorescence microscopy using a DMi8 microscope. Staining with specific mouse cytokeratin antibody and a goat anti-mouse Alexa 488 (green). DSP staining was compared to the ZZ-R127 epithelial cell line, known to strongly express cytokeratin, and the BUcEC endothelial cell line, known to not express cytokeratin. **(C)** Occludin staining. DSP was stained with a specific rabbit occludin antibody and a goat anti-rabbit Alexa 488 (green). DSP staining was compared to the BUcEC endothelial cell line, which is known to not express occludin. **(D)** Vimentin staining. DSP was stained with a specific mouse vimentin antibody and a goat anti-mouse Alexa 488 (green). DSP staining was compared to the ZZ-R127 epithelial cell line, which is known to strongly express cytokeratin. **(E)** α_V_β_6_ integrin staining. DSP was stained with a specific mouse cytokeratin antibody and a goat anti-mouse Alexa 488 (green).

After starting the culture, the DSP cells were observed under a brightfield microscope to analyze their morphology (Figure 3A). We observed cells with a polygonal or cobblestone morphology with somewhat irregular contours, similar to epithelial cells (Yang et al., 2020).

As we did not have enough cells in the culture to fix them for labeling at the beginning of the experiment, we had to wait for several passages before characterizing them by IF. At this point, cells grown in monolayers labeled with an anti-cytokeratin antibody showed a weak signal, drowned in massive autofluorescence compared to the positive control on goat tongue epithelial cells (ZZ-R127). However, no autofluorescence was detected on porcine DSP cells in the absence of primary antibodies (data not shown). Furthermore, the staining performed on bovine endothelial cells derived from the umbilical cord (BUcEC), considered a negative control for cytokeratin detection, was not associated with any signal as shown in Figure 3B.

Labeling to detect occludin, a protein involved in tight junction formation, revealed the expression of occludin in DSP cells, while the secondary antibody alone did not generate fluorescence at similar conditions of fluorescent microscopy (data not shown). Almost no signal was detected on endothelial BUcEC considered as negative control (Figure 3C).

When performed simultaneously, labeling with an anti-vimentin antibody indicated that the cells express more vimentin than cytokeratin in porcine DSP cells (Figure 3D). The secondary antibody alone did not generate fluorescence at similar conditions of fluorescent microscopy (data not shown). Vimentin was also detected in a more diffuse way in the ZZ-R127 epithelial cell line.

Prior to the infection of DSP cells, we wanted to verify that these cells possessed the specific FMDV receptor required for infection initiation, namely, integrin α_V_β_6_. DSP cells were labeled with an antibody raised against this protein. The secondary antibody alone did not generate fluorescence at similar conditions of fluorescent microscopy (data not shown). This labeling enabled us to observe a signal corresponding to a significant expression of the α_V_β_6_ integrin in our cells, as shown in Figure 3E.

### 3.2. Porcine DSPs are susceptible to foot-and-mouth and other vesicular disease viruses

**Figure 4:**
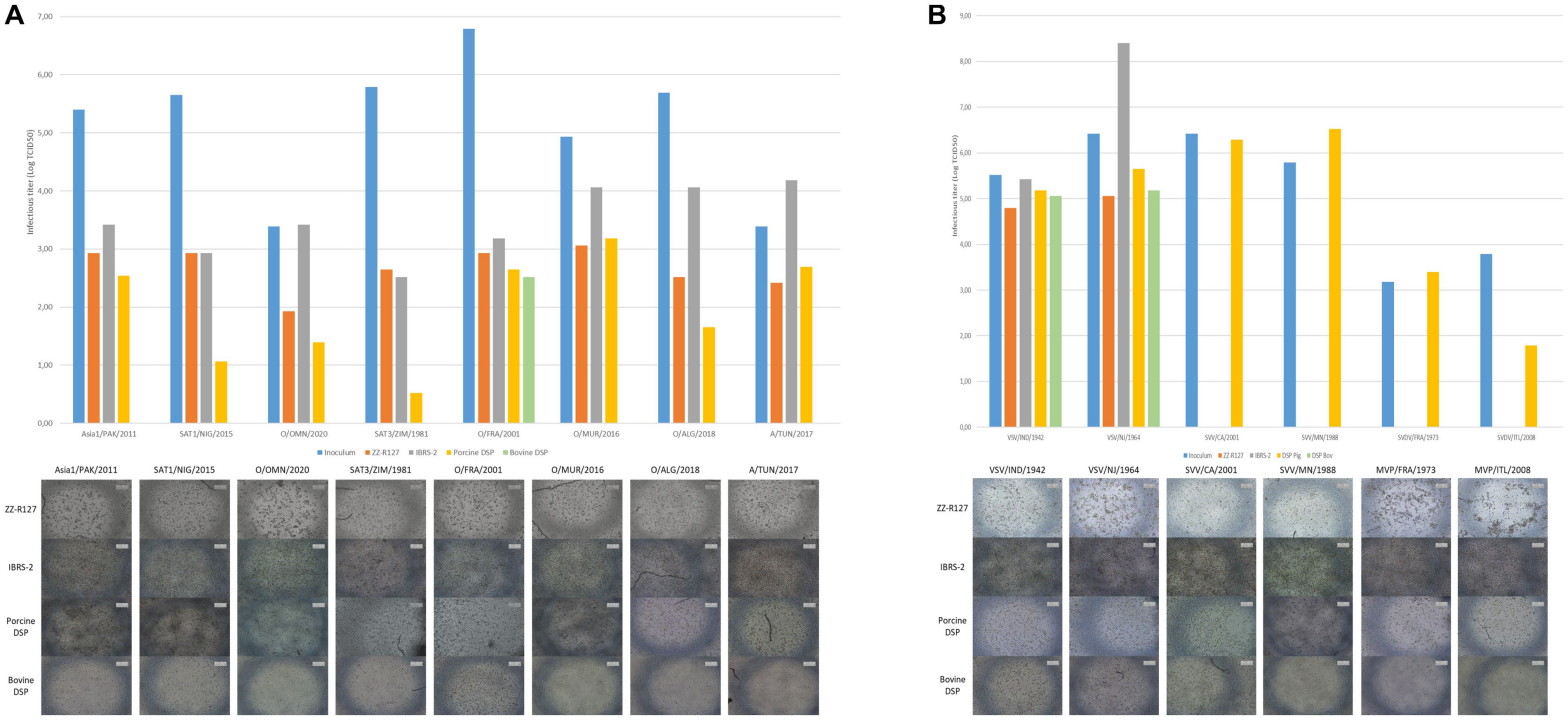
Assessment of porcine DSP susceptibility to a panel of viruses involved in the differential diagnosis of FMDV. **(A)** Porcine DSP susceptibility to FMDV strains. Porcine DSP, bovine DSP, ZZ-R127, and IBRS-2 were cultured in 48-well plates and infected with FMDV Asia1/PAK/2011, SAT1/NIG/2015, SAT3/ZIM/1981, O/OMN/2020, O/FRA/2001, O/MUR/2016, O/ALG/2018, or A/TUN/2017 strain. At 48 h postinfection, CPE was visually determined and supernatants were collected in order to perform viral titration in BHK-21 cells. Virus titers are expressed as TCID50/mL. **(B)** Porcine DSP susceptibility VSV, SVV, and SVDV strains. Porcine DSP, bovine DSP, ZZ-R127, and IBRS-2 were cultured in 48-well plate and infected with VSV/IND/1942, VSV/NJ/1964, SVV/CA/2001, SVV MN/1988, SVDV/FRA/1973, or SVDV/ITL/2008 strain. At 48 h postinfection, CPE was visually determined and supernatants were collected in order to perform viral titration in porcine DSP cells. Virus titers are expressed as TCID50/mL.

Porcine DSP susceptibility to FMDV infection was assessed using eight strains representative of the diversity of reference and field viruses recently detected, namely, Asia1/PAK/2011, SAT1/NIG/2015, SAT3/ZIM/1981, O/OMN/2020, O/FRA/2001, O/MUR/2016, O/ALG/2018, and A/TUN/2017. The infection performance of porcine DSP was compared to ZZ-R127 and IBRS-2 standard diagnostic cell lines, as well as bovine DSP cells (Figure 4A). In contrast to bovine DSP, porcine DSP, like ZZ-R127 and IBRS-2, were shown to be susceptible to all FMDV strains tested, resulting in a systematic CPE at 48 hpi. Titration of corresponding cell culture supernatants showed similar infectious titers between porcine DSP and standard diagnostic cell lines in the case of infection with Asia1/PAK/2011, O/FRA/2001, O/MUR/2016, and A/TUN/2017 strains. However, titers obtained after infection of porcine DSP with SAT1/NIG/2015, SAT3/ZIM/1981, O/OMN/2020, and O/ALG/2018 were significantly lower than those that resulted from ZZ-R127 and IBRS-2 infection (Figure 4B).

**Figure 5:**
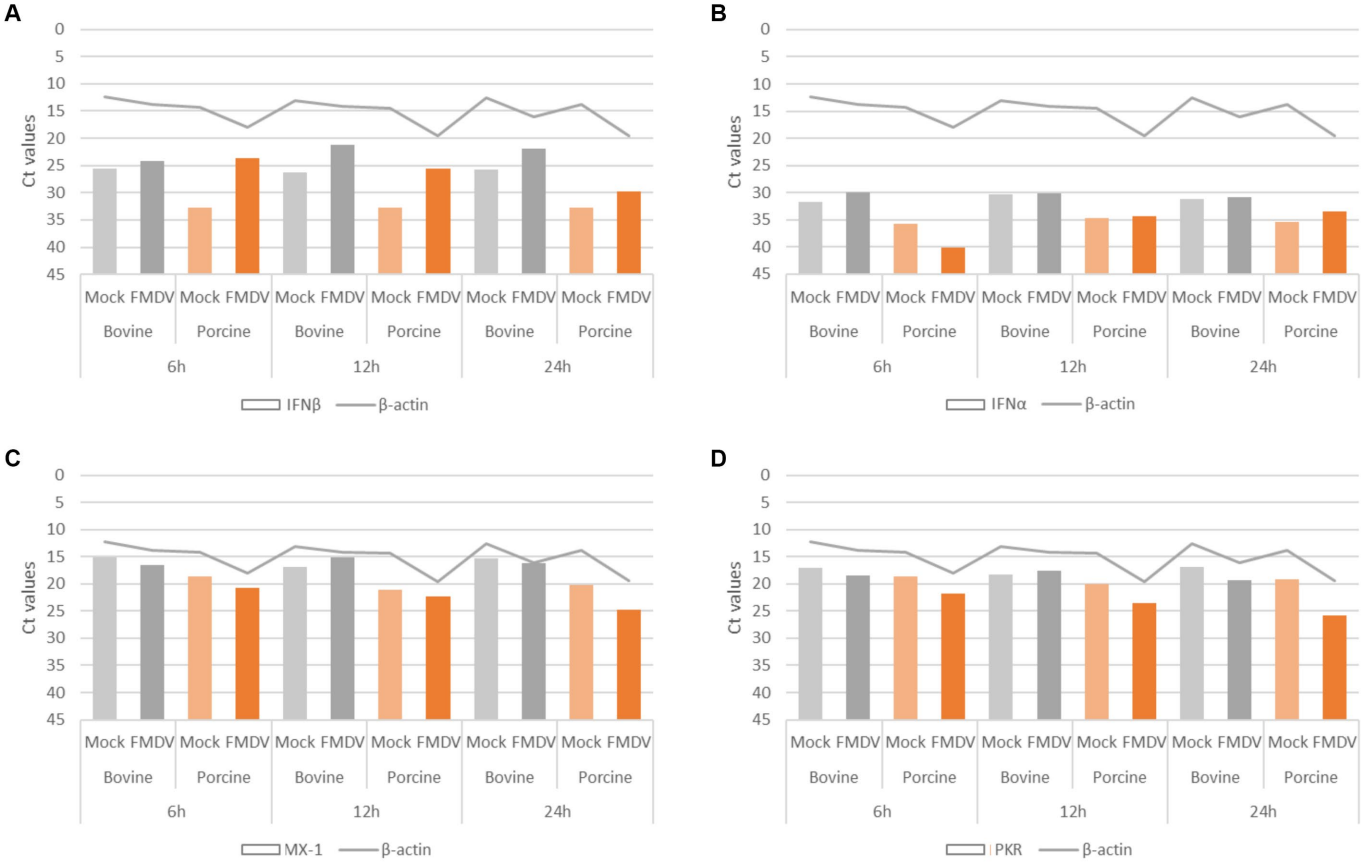
Assessment of porcine DSP ability to produce type-I IFN. Porcine DSP and bovine DSP were cultured in 6-well plate and incubated with FMDV O Cl2.2 or conditioned medium. At 6, 12, and 24 hpi, cellular RNA was extracted in order to perform rtRT-PCR targeting type-I IFN response components. **(A)** IFNβ; **(B)** IFNα; **(C)** MX-1; and **(D)** PKR.

In addition, porcine DSP was tested for susceptibility to viruses involved in the FMD differential diagnosis and compared with the same cells (Figure 5B). Porcine DSP was shown to be susceptible to vesicular stomatitis virus (VSV) strains IND/1942 and NJ/1964, Seneca Valley virus (SVV) strains CA/2001 and MN/1988, and swine vesicular disease virus (SVDV) strains FRA/1973 and ITL/2008. These infection assays revealed a much more pronounced CPE in porcine DSP than in the other cell lines currently used for SVV infection.

### 3.3. Porcine DSP can produce type-I IFN in response to FMDV infection

Porcine DSP’s ability to produce type-I IFN was assessed by rtRT-PCR by comparing RNA expression levels of IFNα, IFNβ, MX-1, and PKR in supernatants from cells infected at MOI 1 or incubated with conditioned medium (Figure 5) at 6, 12, or 24 hpi. Likewise, the IFN response induced by porcine DSP was compared to that induced by bovine DSP in response to FMDV infection at MOI 0.01. The internal control β-actin was detected at cycle threshold (Ct) values between 12 and 20. As porcine DSP exhibited greater CPE than bovine DSP, the highest Ct β-actin values were detected in porcine cell culture supernatants. Consistent with what was observed for bovine cells, IFNβ-associated Ct values decreased significantly when comparing infected porcine DSP supernatants (Ct values between 23 and 29), compared to MOCK samples (Ct values between 32 and 33). The highest induction was observed at 6 hpi for porcine DSP and 12 hpi for bovine DSP, consistent with the higher MOI used during infection. For the IFNα, MX-1, and PKR targets, although no significant changes could be detected between MOCK and infected conditions, Ct values between 33 and 40, 20 and 25, and 21 and 26, respectively, were detected, reflecting cells competent for the type-I IFN pathway.

### 3.4. No infectious FMDV detected beyond 14 days postinfection

**Figure 6:**
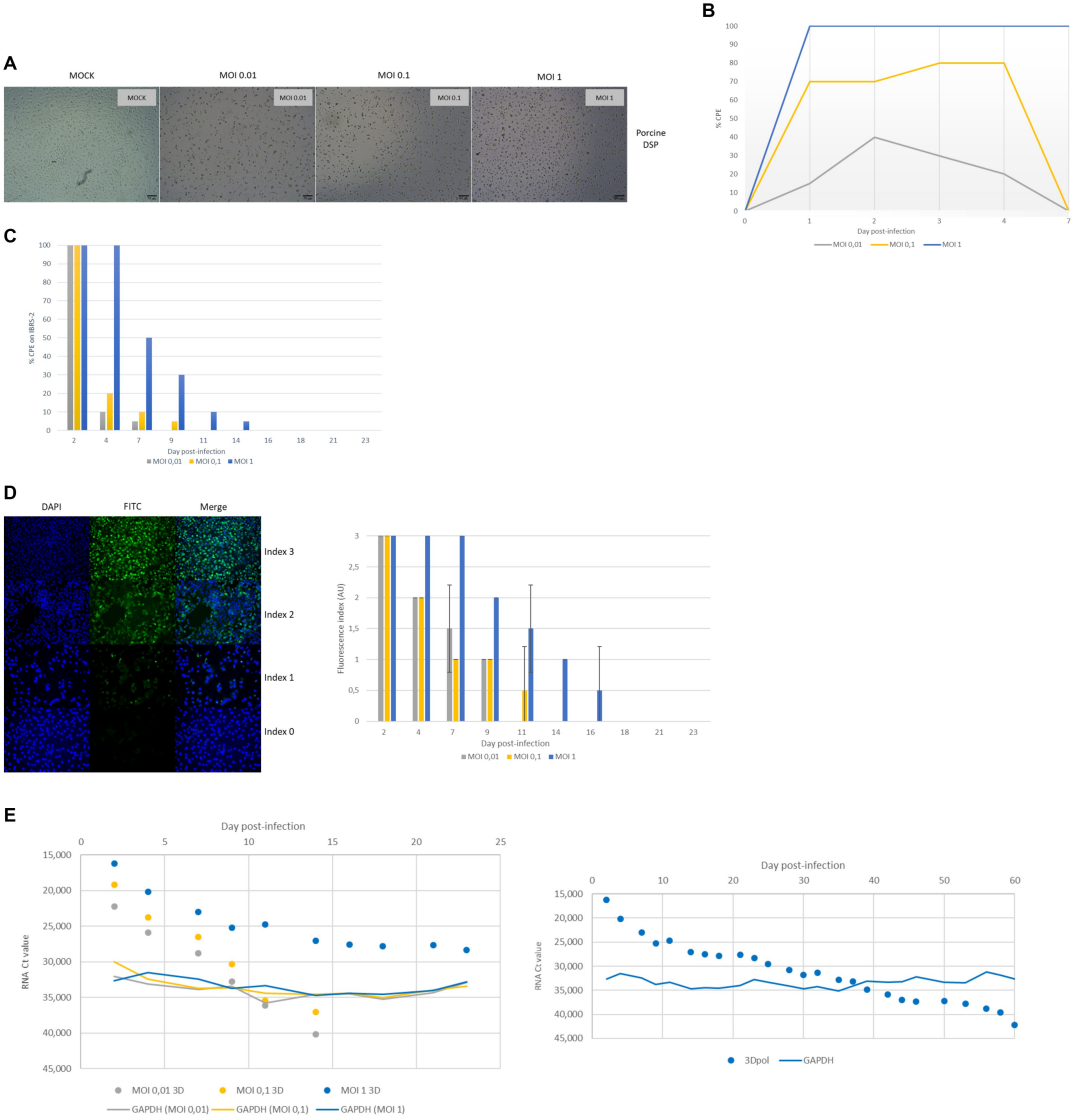
Monitoring of FMDV infection of porcine DSP cultured in monolayers. Porcine DSP was cultured in monolayers and was infected with FMDV O Cl2.2 at MOI 0.01, 0.1, or 1, or a placebo-conditioned medium. **(A)** CPE observation at 48 hpi. **(B)** CPE evolution during the first 7 dpi. CPE was visually estimated using brightfield microscopy. **(C)** Infectious FMDV detection by viral isolation. Viral isolation on IBRS-2 sensitive cells was performed using collected supernatants to detect infectious FMDV. CPE on IBRS-2 after 48 h of incubation with supernatants was visually estimated. The results presented here concern culture supernatants collected during the first 23 days postinfection. **(D)** FMDV 3D^pol^ antigen detection by immunofluorescence. IBSR-2 96-well plates used for viral isolation were fixed with 4% paraformaldehyde and then permeabilized with Triton X-100 before being stained with a specific mouse 3D^pol^ antibody and a goat anti-mouse Alexa 488 (green). Cell nuclei were stained with Hoechst (blue) and cells were visualized by fluorescence microscopy using a DMi8 microscope. Results were arbitrarily classified according to an index ranging from 0 to 3. 0 indicates no fluorescence, 1 indicates a small number of fluorescent cells, 2 indicates a majority of fluorescent cells, and 3 indicates that almost all cells are fluorescent. Evolution of fluorescence levels estimated after 48 h of incubation of IBRS-2 with culture supernatants harvested up to 23 days postinfection. The results presented here are the average of two samples. **(E)** FMDV 3D^pol^ RNA detection by rtRT-PCR. Duplex rtRT-PCR targeting 3D^pol^ FMDV RNA as well as GAPDH housekeeping gene was performed. The results presented here concern results from culture supernatants collected during the first 60 days postinfection.

According to light microscopy observation, FMDV O/FRA/1/2001 Clone 2.2 infection of DSP monolayers resulted in variable cell lysis depending on both the MOI used during infection (Figure 6A). Indeed, CPE was detectable between 1 and 4 dpi for the three MOI tested, i.e., 1, 0.1 (Figure 6B). While the estimated CPE was close to 100% at MOI 1, it did not exceed 80% at MOI 0.1 and 40% at MOI 0.01. No CPE was detected for the MOCK samples. After 7 dpi, whatever the MOI used, the infected cells that were still alive managed to grow again and to reform pieces of cell monolayers of varying size. This was also true for MOI 1, for which we estimated a CPE close to 100%, indicating that there were still a few cells that survived the infection. The reconstruction of the cell monolayers was very gradual and was confronted with new events of cell degradation, especially during the first 10 days after infection (data not shown). Recovery of a complete monolayer required 4 days for DSP infected at MOI 0.01, 7 days for cells infected at MOI 0.1, and 24 days for cells infected at MOI 1.

Cell culture supernatants collected during the whole experiment were tested for the presence of infectious viruses by inoculation on FMDV-sensitive IBRS-2 cells. FMDV-induced CPE on IBRS-2 was estimated by microscopy observation and is summarized in Figure 6C. Under our experimental conditions, no CPE was detected for the MOCK samples. Again, results depended on both the MOI used for the infection and the infected cell population. Thus, CPE, which revealed infectious FMDV particles were present, was detected in supernatants collected up to 14 dpi for DSP infected at MOI 1 and up to 9 and 7 dpi, respectively, for the cells infected at MOI 0.1 and 0.01.

Porcine cells IBRS-2 96-well plates used for viral isolation were then fixed and stained to detect FMDV antigens, in particular, 3D^pol^ antigens by IF (Figure 6D). No specific fluorescence was found to be associated with MOCK conditions. Again, results depended on both the MOI used for the infection. Indeed, 3D^pol^ antigens were detected in IBRS-2 incubated with DSP samples collected up to 14 dpi for MOI 1, 11 dpi for MOI 0.1, and 9 dpi for MOI 0.01.

Throughout the experiment, cell culture supernatants were analyzed for the presence of viral RNA by rtRT-PCR targeting the 3D^pol^ protein coding region of FMDV. The internal control GAPDH was detected at Ct values between 29 and 37. FMDV RNA was not detected in the cell culture supernatant prior to infection but was detected after DSP infection at MOI 1, 0.1, and 0.01 (Figure 6E). FMDV 3D^pol^ RNA was detected by rtRT-PCR in supernatants collected up to 14 dpi for cells infected at MOI 0.01 (Ct values between 22 and 40) and 0.1 (Ct values between 19 and 37) and up to 60 dpi for the MOI 1 (Ct values between 16 and 42).

### 3.5. Primary porcine DSP cells grew in multiple layers at the air-liquid interface

**Figure 7:**
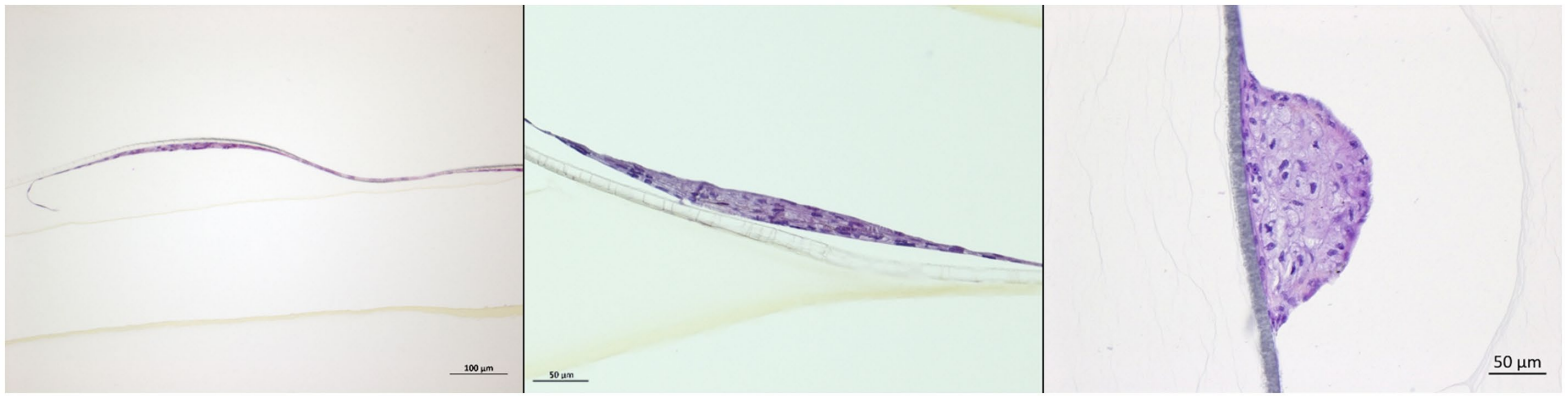
DSP cells multilayer characterization. Porcine DSP was cultured in multilayers at the air-liquid interface for 6 weeks. Some inserts were sacrificed to evaluate the multilayer depth. To this purpose, inserts were fixed in formalin and ethanol and then stained with HES. Stained inserts were observed by microscopy to estimate the number of cell layers.

In order to study the cooperation that may exist between the layers forming an epithelium, DSP multilayers cultured at the ALI were grown and then infected. Infection was monitored in the same manner that was used to monitor infection on monolayers. As revealed by HES staining of the inserts, after 5 weeks in the culture at the ALI, the DSP was organized into a layer dotted with a multitude of cell clusters composed of a few layers of cells (Figure 7).

### 3.6. No infectious FMDV detected beyond 7 dpi on porcine DSP multilayers

**Figure 8:**
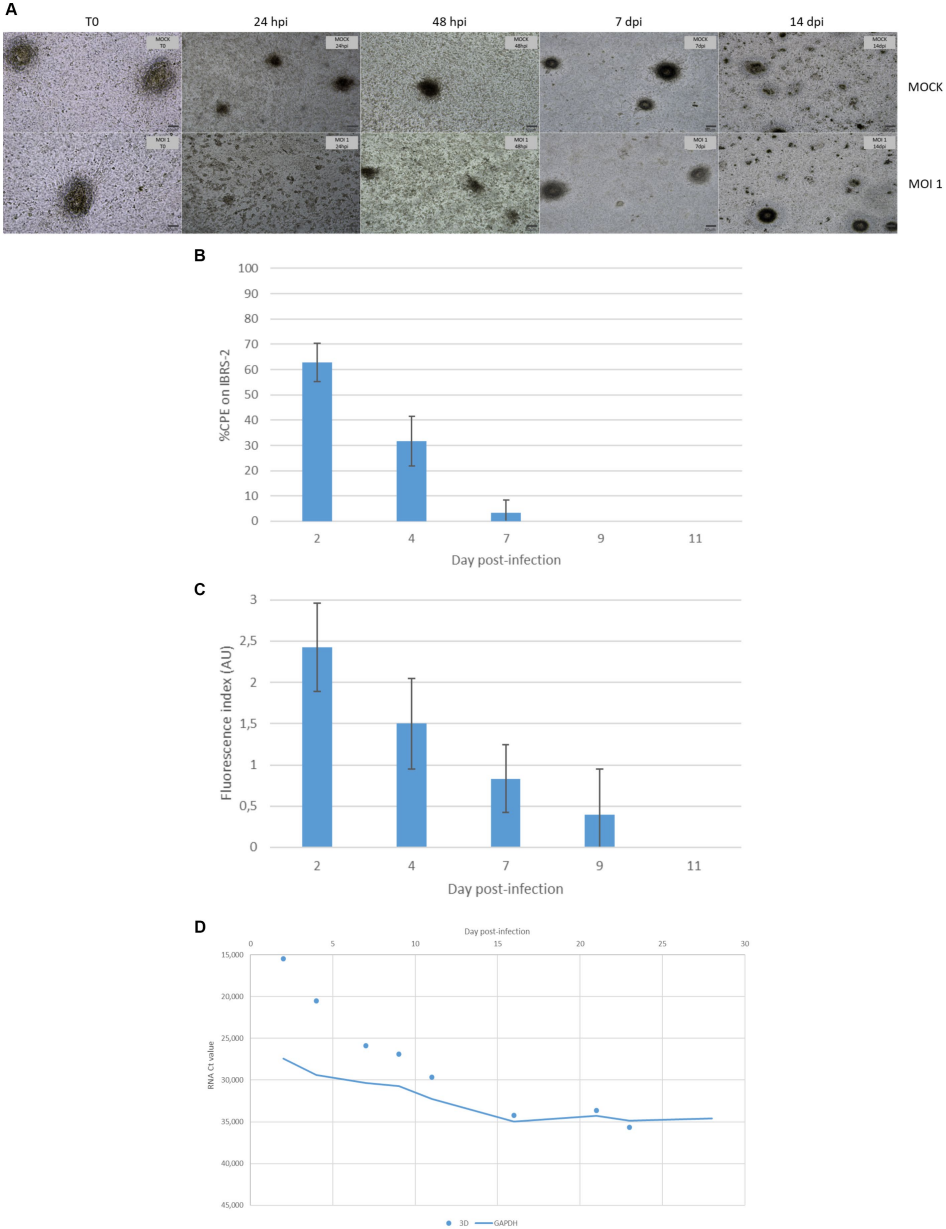
Monitoring of FMDV infection of porcine DSP cultured in multilayers at the air-liquid interface. DSP was cultured in multilayers at the air-liquid interface for 6 weeks before being infected with FMDV O Cl2.2 at MOI 1, or a placebo-conditioned medium. CPE was visually estimated almost daily during the first 2 weeks postinfection. **(A)** CPE evolution on DSP multilayers. Microscopic CPE observation was pictured just before infection, at 24 hpi, 48 hpi, 7 dpi, and 14 dpi for infected and MOCK conditions. **(B)** Infectious FMDV detection by viral isolation. Viral isolation on IBRS-2 sensitive cells was performed using collected supernatants to detect infectious FMDV. CPE on IBRS-2 was visually estimated after 48 h of incubation with supernatants passaged once in IBRS-2. The results presented here concern culture supernatants collected during the first 11 days postinfection. **(C)** FMDV 3D^pol^ antigen detection by immunofluorescence. IBSR-2 96-well plates used for viral isolation were fixed with 4% paraformaldehyde and then permeabilized with Triton X-100 before being stained with a specific mouse 3D^pol^ antibody and a goat anti-mouse Alexa 488. Cell nuclei were stained with Hoechst and cells were visualized by fluorescence microscopy using a DMi8 microscope. Results were arbitrarily classified according to an index ranging from 0 to 3. 0 indicates no fluorescence, 1 indicates a small number of fluorescent cells, 2 indicates a majority of fluorescent cells, and 3 indicates that almost all cells are fluorescent. Here, the evolution of fluorescence levels estimated after 48 h of incubation of IBRS-2 with culture supernatants harvested up to 11 days postinfection is presented. The results presented here are the average of two samples. **(D)** FMDV 3D^pol^ RNA detection by rtRT-PCR. Duplex rtRT-PCR targeting 3D^pol^ FMDV RNA as well as GAPDH housekeeping gene was performed. The results presented here concern results from culture supernatants collected during the first 28 days postinfection.

According to light microscopy observation, the FMDV O/FRA/1/2001 Clone 2.2 infection of DSP multilayers resulted in a slight CPE that was difficult to estimate. Indeed, the addition of medium to the inserts at the time of infection resulted in significant desquamation of the multilayers in the first 2 days postinfection, which made the visualization of the FMDV-mediated CPE less apparent. However, cell clusters and the monolayer appeared to be more severely affected in the infected inserts than in the MOCK, indicating weak FMDV-induced cell lysis as early as 24 hpi (Figure 8A). Differences between the infected and MOCK inserts remained observable until 10 dpi, without further cell lysis. The cultures seemed to have quickly recovered before 14 dpi.

Cell culture supernatants collected during the whole experiment were tested for the presence of infectious viruses on IBRS-2 cells. After 48 hpi, no CPE was detected on IBRS-2. Therefore, we decided to perform a second passage of all supernatants on IBRS-2. No CPE was detected for the MOCK samples. As shown in Figure 8B, CPE was detected for upper supernatants collected up to 7 dpi after being passaged once on IBRS-2. The IBRS-2 96-well plate used for the second viral isolation was then fixed and stained to detect 3D^pol^ antigens (Figure 8C). No specific fluorescence was found to be associated with MOCK conditions. Viral antigen-positive cells were observed when investigating supernatants harvested up to 9 dpi.

Insert upper supernatants were analyzed for the presence of viral RNA by rtRT-PCR targeting the 3D^pol^ protein coding region of FMDV. The internal control GAPDH was detected at Ct values between 25 and 35. FMDV RNA was not detected in the insert upper supernatant prior to infection. 3D^pol^ RNA was detected by rtRT-PCR in supernatants collected up to 23 dpi as shown in Figure 8D. The measured Ct values ranged from 15 for samples collected at 2 dpi to over 35 for those collected at 23 dpi. No signal corresponding to 3D^pol^ RNA could be detected in the samples collected beyond 23 dpi.

## 4. Discussion

### 4.1. Facing a lack of relevant *in vitro* models for FMDV infection

While BTY cells have been considered the most susceptible cells to FMDV infection for a long time, the constraints associated with their culture have resulted in the use of cell lines such as BHK-21, IBRS-2, and ZZ-R127 (Brehm et al., 2009). Except for the latter, derived from goat tongue, none of the commonly used cell lines are derived from tissues relevant to the study of FMDV. Since none of the porcine cell lines used for FMDV research and diagnosis derive from relevant locations, this study describes the development of a porcine DSP cell model particularly suited to this virus.

Primary epithelial cells from porcine oropharyngeal tonsils and DSP were isolated and then cultured. While cells from the DSP, described as one of the primary sites of FMDV replication, were successfully cultured, we were unable to culture cells from the oropharyngeal tonsils, a structure considered to be the most important site of primary and sustained FMDV replication (Stenfeldt et al., 2014). Indeed, cells from the oropharyngeal tonsils of three pigs had difficulty adhering to the culture flask and did not survive trypsinization. As the collection and culture protocol was followed in exactly the same way for both cell populations, we concluded that these cells required an additional factor to allow the adhesion and/or detachment of the few cells that adhered to their support. Unfortunately, this factor could not be identified in the framework of this study. The difference observed in the behavior of DSP cells and oropharyngeal tonsil cells could also be due to the number of epithelial cells available in the starting samples. Only a few studies concerning pig oropharyngeal tonsil cell culture have been reported in the literature, and most of them did not deal with epithelial cells but with myeloid or lymphoid cells (Razzuoli et al., 2012, 2014; Soldevila et al., 2018). To the best of our knowledge, only one research paper referred to the establishment of a pig tonsil epithelial cell line (Xi et al., 2020). However, this article did not mention the use of a particular factor in addition to a classical culture medium DMEM/F12 with FCS and did not help us to maintain these cells in culture.

### 4.2. Porcine DSP are epithelial cells able to produce type-I IFN

Regarding the DSP-derived cells, we were able to identify the presence of epithelial tissue in the explants, culture these cells, maintain them, and perform freeze–thaw resistance assays. Characterization of DSP cells enabled us to confirm their epithelial nature. Indeed, although they weakly express cytokeratin, an important epithelial marker, in comparison with epithelial cells from the ZZ-R127 lineage, we observed cells with a characteristic epithelial cell polygonal morphology (Wang et al., 2012; Kuburich et al., 2022). Furthermore, we showed that these cells constitutively express occludin, a protein involved in tight junction formation in epithelia (Matter and Balda, 1999). The low expression of cytokeratin in our cells is consistent with the high detection of vimentin, a cytoskeletal intermediate filament protein characteristic of fibroblastic cells but which can also be found in epithelial cells, and potentially involved in FMDV survival (Gladue et al., 2013). Generally, vimentin production has been reported to be associated with the epithelial-mesenchymal transition (EMT), a highly dynamic and reversible biological process by which an epithelial cell undergoes biochemical changes that allow it to adopt a mesenchymal cell phenotype (Guarino, 2007; Kalluri and Weinberg, 2009; Yang et al., 2020). As this transition is known to affect primary epithelial cells over passages, it is not surprising that vimentin is detected to a greater extent than cytokeratin as these markings were performed after seven passages (Pieper et al., 1992).

We have also demonstrated the ability of porcine DSP to trigger an innate immune response during infection. As part of this study, we focused on the type-I IFN response, the first line of defense against pathogens, known to be significantly activated during FMDV infection (Ma et al., 2018; Sarry et al., 2022). We identified a substantial increase in the RNA associated with IFNβ expression level in response to FMDV infection. Although the absolute expression levels are slightly lower than those measured in bovine DSP, the activating effect following FMDV infection appears to be more pronounced in porcine DSP. Despite no significant increase in the expression levels of RNA corresponding to IFNα, as well as MX-1 and PKR ISG, within 24 hpi, high basal expression levels similar to those found in bovine DSP were measured, suggesting that the cells were competent in their type I IFN pathway.

### 4.3. Porcine DSP is susceptible to foot-and-mouth and other vesicular disease viruses

Anti-integrin α_V_β_6_ antibody cell staining revealed expression of this protein, considered to be the preferential receptor for FMDV (Monaghan et al., 2005). We therefore expected that DSP would be susceptible to this virus. Infection assays performed in monolayers confirmed this assumption.

Indeed, we demonstrated the susceptibility of porcine DSP to a panel of eight FMDV strains representative of the diversity of reference and field strains detected in recent years, namely, Asia1/PAK/2011, SAT1/NIG/2015, SAT3/ZIM/1981, O/OMN/2020, O/FRA/2001, O/MUR/2016, O/ALG/2018, and A/TUN/2017. Although the viral titers obtained 48 hpi in the porcine DSP were slightly lower overall than those obtained after infection of the standard diagnostic cell lines ZZ-R127 and IBRS-2, significant CPE appeared following infection of the porcine DSP, making it easy to detect the infection.

Likewise, infection of porcine DSP with VSV IND/1942 and NJ/1964 strains, SVV CA/2001 and MN/1988 strains, and SVDV FRA/1973 and ITL/2008 strains highlighted the susceptibility of these cells to the viruses involved in the FMDV differential diagnosis. In addition, infected porcine DSP showed an important CPE resuting in round and floating cells. This is also true for SVV infection, a virus known in our experience to induce only moderate CPE on the lineage cells currently used for diagnosis.

Regarding our preliminary assays for long-term infection, we found that these porcine cells were less sensitive to FMDV O/FRA/1/2001 Clone 2.2 than the bovine DSP cultured by Hägglund et al. (2020). Indeed, whereas the bovine DSP infected at MOI 0.01 showed an almost complete CPE at 24 hpi, the porcine DSP infected at the same MOI showed less than 20% CPE. To reach an almost complete CPE at 24 hpi the porcine DSP had to be infected at MOI 1. Obtaining and infecting epithelial cells from oropharyngeal tonsils would have been useful to determine whether porcine epithelial cells from tissues of interest are all less susceptible than bovine cells or whether those from tonsils, considered as the main site of primary replication, are more susceptible to FMDV (Stenfeldt et al., 2014). Given the results obtained when assessing the IFN response in porcine and bovine DSP, it would not be possible to explain the lower sensitivity of porcine cells by a stronger type-I IFN response. However, it is not excluded that other immune response pathways are activated to a greater extent.

Regardless of the MOI used, we were able to detect infectious FMDV in the culture supernatants by viral isolation on susceptible IBRS-2 cells. Infectious viruses were detected up to 14 dpi in the supernatants of DSP infected at MOI 1. Infectious viruses were associated with viral antigens, notably 3D^pol^, which we were able to detect up to 16 dpi in the supernatants of DSP infected at MOI 1, as well as viral RNA. Although rtRT-PCR detection of viral RNA was no longer possible beyond 14 dpi for cells infected at MOI 0.01 and 0.1, we were able to identify RNA corresponding to the FMDV 3D^pol^ up to 60 dpi in supernatants from MOI 1 infection. RNA Ct values detected several weeks after termination of infectious virus detection are over 35 Ct and increase over time. Such low RNA levels could potentially be related to the generation of a limited quantity of defective viral particles or to the antiviral cellular response-mediated FMDV inhibition (Arzt et al., 2017; Stenfeldt et al., 2017; Pfaff et al., 2019).

### 4.4. Infection of porcine DSP multilayers does not indicate the existence of FMDV persistence

Porcine DSP cells were cultured at the air-liquid interface in order to reproduce a stratified epithelium, mimicking a natural epithelium, inspired by the work carried out on bovine DSP by Hägglund et al. (2020). The histological characterization of our multilayer cultures confirms the establishment of a stratified epithelium. However, it appears that similar to the bovine model, our model does not allow the reconstitution of the epithelium as deep as those observed *in vivo* (Schley et al., 2011).

After infection with FMDV at MOI 1, DSP cells grown in multilayers showed a very limited CPE during the first few days postinfection, similar to what was observed with the bovine model infected at MOI 0.01. The desquamation of the cells upon contact with the inoculum did not however facilitate the observation of CPE as a small proportion of the cells also detached from the multilayers for the MOCK conditions. The limited CPE resulted in a rapid reconstitution of the cell layers after less than 14 dpi. As discussed by Hägglund et al. (2020), this limited CPE is in line with the absence of lesions and low viral replication that can be observed *in vivo* in DSP (Zhang and Alexandersen, 2004; Murphy et al., 2010; Stenfeldt and Belsham, 2012; Stenfeldt et al., 2014, 2016c; Hägglund et al., 2020). Furthermore, Hägglund et al. (2020), highlighted that the difference in susceptibility found between DSP cells grown in monolayers and those grown in multilayers could be correlated to the presence of integrin α_V_β_6_ in monolayers but not in multilayers within which FMDV could enter via other receptors such as other integrins α_V_β_1_, α_V_β_3_, and α_V_β_8_ or heparan sulfate (Jackson et al., 1996; Baranowski et al., 2000; O’Donnell et al., 2008, 2014).

The limited CPE observed during multilayer infection may also be a consequence of the genome distribution of FMDV, which is concentrated in the basal layer, whereas the viral antigens are concentrated in the superficial layer of the soft palate epithelium, thus limiting the CPE visible on the surface (Stenfeldt et al., 2016a). Analysis of upper culture supernatants collected during infection of the multilayer model enabled us to demonstrate the presence of infectious FMDV up to 7 dpi. However, the detection of infectious viruses was only possible after a second passage of the supernatants on IBRS-2 sensitive cells, indicating that the viral load present in the harvested culture supernatants was too low to induce a visible CPE on these cells within 48 h. Again, the distribution of infectious viruses in the deep layers of the epithelium could be one of the possible causes for the low viral load detected in the supernatants collected from the upper surface. Staining of the IBRS-2 96-well plate used for the detection of infectious viruses allowed us to confirm that the observed CPE was indeed related to FMDV, as we were able to detect 3D^pol^ viral antigens in cells incubated in the presence of culture supernatants collected up to 9 dpi. Furthermore, we showed by rtRT-PCR that the collected supernatants contained 3D^pol^ RNA up to 23 dpi. All these results tend to show that the infection of porcine DSP cultured in multilayers only led to weakly productive infection, of short duration and probably rapidly countered by the antiviral cellular response, corroborating the *in vivo* observations that showed a decrease in the quantity of FMDV detected over time and a viremia phase lasting only a few days in pigs (Arzt et al., 2011a; Stenfeldt et al., 2014, 2016c).

The main parameters controlled during the infection of the porcine cell model developed in this study have been presented in Table 1 and compared with those published by Hägglund et al. (2020) regarding the bovine model.

**Table 1:** Comparative analysis of FMDV O Cl2.2 infection monitoring of porcine DSP cells cultured in monolayers and multilayers at the air-liquid interface as well as bovine DSP cells cultured in multilayers at the air-liquid interface.

	Susceptibility to FMDV infection	Time to complete destruction of the cell layers	Time to complete cell layer recovery	3D^pol^ RNA detection (up to)	Infectious virus detection (up to)	3D^pol^ antigen detection (up to)
Porcine DSP Monolayers	++++	1 dpi	25 dpi	60 dpi	14 dpi	16 dpi
Porcine DSP Multilayers	+	X	14 dpi	23 dpi	7 dpi	9 dpi
^*^Bovine DSP Multilayers	++	X	28 dpi	>28 dpi	>28 dpi	>28 dpi

^*^The results for pig cells are entirely based on this study, while the results for bovine cells are based on the work of Hägglund et al. (2020).

## 5. Conclusion and perspectives

In this study, we demonstrated that FMDV infections of porcine DSP cells grown as monolayers and multilayers at the air-liquid interface are consistent with those *in vivo.* Accordingly, since no infectious virus was detected beyond 14 dpi, there was no evidence of FMDV persistence. Compared to the currently used porcine cell models, this new model of cells cultured at the air-liquid interface, unaffected by cell passages, allows for the reproduction of natural conditions by mimicking a stratified epithelium sensitive to FMDV infection. It can thus be used alongside the bovine model developed by Hägglund et al. (2020) to further investigate the differential persistence of FMDV, with particular reference to the conservation of epithelial innate immune responses from one species to another during infection. By allowing hypotheses to be tested in biological models that are more suitable than monolayer cultured cells, the use of these multilayer models will reduce the number of experiments involving animals. The porcine primary DSP cells cultured in this study could also be immortalized to produce the first tissue-derived cell line of interest for the study of FMDV. Such a cell line could be used for both FMDV research and diagnosis. Given their great susceptibility to SVV, VSV, and SVDV infection, these cells could be considered as a useful tool for swine viral vesicular disease differential diagnosis.

## Data availability statement

The raw data supporting the conclusions of this article will be made available by the authors, without undue reservation.

## Ethics statement

Ethical approval was not required for the study involving animals in accordance with the local legislation and institutional requirements because tissue sampling was carried out on pigs that had already been euthanised as part of another project validated by the ethical committee of the Maisons-Alfort veterinary school.

## Author contributions

SB-B, LBK, J-FV, and MS: conceptualization. J-FV, SH, SB-B, and MS: methodology. MS, SB-B, CB-C, CM, ARe, ARo, A-LS, PR, MC, MB, HH, and GJ: investigation. MS: original draft preparation. SB-B and LBK: supervision. SB-B, LBK, J-FV, SH, CB-C, CM, and ARo: reviewing. All authors contributed to the article and approved the submitted version.
